# Interleukin-38 is a negative regulator of trained immunity—A retrospective multi-omics study

**DOI:** 10.1016/j.isci.2025.113758

**Published:** 2025-10-14

**Authors:** Lisa U. Teufel, Vasiliki Matzaraki, Lukas Folkman, Dennis M. de Graaf, Rob ter Horst, Simone J.C.F.M. Moorlag, Jéssica C. dos Santos, Catharina M. Mulders-Manders, Thomas Krausgruber, Charles Dinarello, Mihai G. Netea, Leo A.B. Joosten, Rob J.W. Arts

**Affiliations:** 1 Department of Medical BioSciences, Research Institute for Medical Innovation, Radboud University Medical Centre, Nijmegen, the Netherlands; 2 Department of Internal Medicine and Radboudumc Center for Infectious Diseases (RCI), Radboud University Medical Centre, Nijmegen, the Netherlands; 3 Department of Immunology and Metabolism, Life and Medical Sciences Institute, University of Bonn, Bonn, Germany; 4 Department of Medical Genetics, Iuliu Hatieganu University of Medicine and Pharmacy, 400349 Cluj-Napoca, Romania; 5 CeMM Research Center for Molecular Medicine of the Austrian Academy of Sciences, Vienna, Austria; 6 Medical University of Vienna, Institute of Artificial Intelligence, Center for Medical Data Science, Vienna, Austria; 7 Department of Medicine, University of Colorado, Aurora, CO 80045, USA; 8 Institute of Innate Immunity, Medical Faculty, University of Bonn, Bonn, Germany

**Keywords:** Immunity, Immune response, Omics

## Abstract

Trained immunity is a long-lasting innate immune cell phenotype with benefits in infection control and recognized anti-cancer effects. Conversely, inappropriately induced trained immunity contributes to pathological inflammation, warranting the exploration of regulatory pathways. We explore interleukin-38 (IL-38) as a regulator of trained immunity *in vivo* in a cohort of 325 healthy adults vaccinated with Bacillus Calmette-Guérin (BCG). Using multi-omics profiling, we find that IL-38 is negatively associated with trained immunity on metabolic and epigenetic level. Genetic variants in *IL1F10*, encoding for IL-38, further link IL-38 to diminished training responses. These associations were validated in human and murine models. We confirmed that IL-38 functionally impairs anti-microbial traits of trained immunity in trained immunity-infection models *in vivo* (IL-38KO mice) and *in vitro* (human monocytes). Our study therefore suggests that IL-38 endogenously regulates the induction of trained immunity in humans *in vivo*.

## Highlights

• IL-38 inhibits all hallmarks of trained immunity in humans
• Plasma IL-38 concentrations regulate BCG-induced trained immunity in humans
• IL-38 inhibits bone-marrow trained immunity in mice
• IL-38 lowers trained immunity-based protection from fungal and parasitic infections

## Graphical abstract

**Figure:**
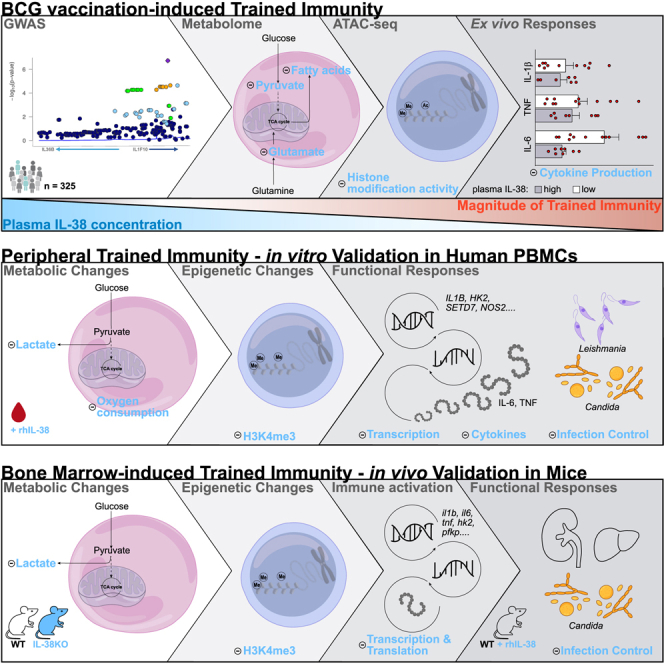

## Introduction

Trained immunity is a hyperresponsive status of innate immune cells including tissue-resident macrophages,^1^ dendritic cells (DCs), monocytes, natural killer (NK) cells, and their hematopoietic stem and progenitor cells,^2^^,^^3^^,^^4^ which is induced through metabolic^5^^,^^6^ and genome-wide epigenetic modifications.^3^^,^^7^^,^^8^^,^^9^ Next to increased myelopoiesis and local cell expansion of innate cell populations at bone marrow level,^10^ enhanced metabolic activity and increased non-specific reactivity are characteristic for trained immunity.^11^ To date, various compounds are known to induce this functional reprogramming, including vaccines (e.g., Bacillus Calmette-Guérin [BCG]^11^), pathogens (e.g., *Plasmodium falciparum*^12^), their ligands (e.g., fungal β-glucan^11^), metabolic intermediates (e.g., oxidized low-density lipoprotein [oxLDL],^11^ uric acid,^13^ and fumarate^5^), and inflammatory cytokines (e.g., interleukin [IL]-1β^9^ and IL-36γ^14^).

The beneficial traits of trained immunity to protect against bacterial, fungal, viral, and parasitic infections^15^^,^^16^^,^^17^ as well as a link to efficient anti-cancer immune responses^18^^,^^19^ are increasingly well understood. Conversely, maladaptive trained immunity as contributor to the pathophysiology of autoinflammatory diseases (hyper-IgD-syndrome (HIDS)^20^) and to chronic inflammation (atherosclerosis^21^^,^^22^ and gout^23^^,^^24^) is only an emerging concept. Based on IL-1 as driver of trained immunity and contributor to autoinflammation,^25^^,^^26^ regulatory members of the IL-1 family are potential endogenous regulators of trained immunity and therefore maladaptive inflammation.

To date, all anti-inflammatory cytokines of this group—IL-1 receptor antagonist (Ra), IL-36Ra, IL-37, and IL-38—have been described in the context of trained immunity *in vitro* or in murine models.^14^^,^^27^^,^^28^^,^^29^ We previously identified a genetic variant in *IL1F10* (coding for IL-38) associated with dampened trained immunity responses in humans.^28^ Yet, it has not been proven that IL-38 protein dampens trained immunity responses nor that IL-38 protein regulates the induction of trained immunity in humans on metabolic and epigenetic level.

We hypothesize that IL-38 inhibits the induction of trained immunity systemically and propose that healthy individuals with high IL-38 plasma concentrations have impaired training responses with possible consequences for infection control. To this end, we characterize pathways underpinning trained immunity inhibition by IL-38 and elucidate the effect of IL-38 protein on fungal and parasitic infection control in human *in vitro* and murine *in vivo* models of trained immunity.

## Results

### IL-38 regulates trained immunity in human monocytes *in vitro*

**Figure 1:**
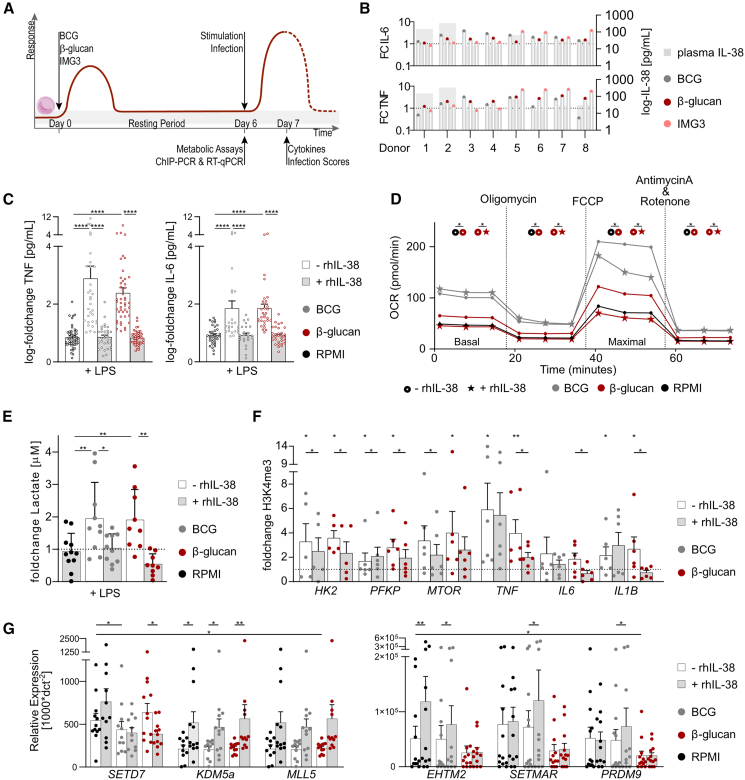
RhIL-38 regulates trained immunity responses in human monocytes (A) Overview of *in vitro* training experiments. (B) Endogenous plasma IL-38 concentrations do not correlate with trained immunity responses. Cells trained with β-glucan (1 μg/mL; red), BCG (5 μg/mL; gray), or *L. braziliensis* (IMG3) (25 μg/mL; rose) were restimulated with LPS (1 ng/mL). IL-6 and TNF were measured after 24 h. Plasma IL-38 concentrations were assessed by ELISA (gray); *n* = 8. (C) Trained immunity induction *in vitro* is inhibited in the presence of rhIL-38. Cells were pre-incubated with rhIL-38 (90 ng/mL), trained with β-glucan (1 μg/mL; red) or BCG (5 μg/mL; gray), and restimulated with LPS (1 ng/mL). IL-6 and TNF were measured after 24 h; *n* = 32–47. Data are presented as mean ± SD. (D) Extracellular flux analyses show partly increased oxygen consumption rate (OCR) in trained cells, which is trainer-dependently inhibited by rhIL-38 (star-shaped); *n* = 6. Data are shown as ± SEM. (E) Trained monocytes (β-glucan [1 μg/mL; red], BCG [5 μg/mL; gray]), restimulated with LPS (1 ng/mL), have elevated lactate production, which is inhibited by rhIL-38 (90 ng/mL; grey-filled); n = 9–10. Data are presented as mean ± SD. (F) Histone 3 lysine 4 trimethylation (% of total input) is elevated in trained cells (β-glucan [1 μg/mL; red], BCG [5 μg/mL; gray]), which is opposed by rhIL-38 (90 ng/mL; grey-filled); *n* = 6. Data are presented as mean ± SEM. (G) RhIL-38 (90 ng/mL; grey-filled) affects the expression of chromatin modifiers in trained cells (β-glucan [1 μg/mL; red], BCG [5 μg/mL; gray]); *n* = 9–12. Data are presented as mean ± SEM. Data (C), (D), (E), (F), and (G) were analyzed by one-tailed Wilcoxon matched-pairs signed rank test; ∗*p* < 0.05, ∗∗*p* < 0.01, ∗∗∗*p* < 0.001, and ∗∗∗∗*p* < 0.0001. See also Data S1.

We studied recombinant human (rh) IL-38 as a potential regulator of trained immunity, assessing cytokine and microbicidal responses, cellular metabolism, and epigenetic signatures in human monocytes *in vitro* (Figure 1A). As endogenous IL-38 concentrations in the culture medium were below 1% of the rhIL-38 concentration used *in vitro*, with minimal variability between experiments, plasma IL-38 was excluded as confounding factor. Further, donor variation did not correlate with the magnitude of trained immunity assessed by cytokine response upon secondary stimulation (Figure 1B) (Spearman correlation; BCG r_TNF_(6) = 0.000, *p* = 1.000, r_IL-6_(6) = 0.024, *p* = 0.979; β-glucan r_TNF_(6) = 0.024, *p* = 0.979, r_IL-6_(6) = −0.049, *p* = 0.928; IMG3 r_TNF_(6) = 0.024, *p* = 0.979, r_IL-6_(6) = −0.244, *p* = 0.564).

We consistently found lower IL-6 and tumor necrosis factor (TNF) production in response to secondary stimulation by cells trained in the presence of rhIL-38 (Figure 1C and Data S1B). We have previously shown that the phosphorylation of serine/threonine kinase mechanistic target of rapamycin (mTOR) and the transcription factor nuclear factor κB, both pivotal for the induction of trained immunity, is reduced by rhIL-38 *in vitro.*^14^^,^^28^ IL-38 has similar effects as IL-36RA, which has been shown to inhibit trained immunity induced by IL-36γ,^14^ upon binding of the IL-36 receptor (IL-36R).^30^ It is postulated that IL-38 binds not only IL-36R but also IL-1R,^30^ although the literature on this matter is conflicting.^31^ As IL-1 is a crucial driver of trained immunity,^25^ we hypothesize that interference of IL-38 with IL-1 signaling may contribute to the regulatory effect on trained immunity (Data S1C).

Previous studies show that a training stimulus-dependent increase in metabolic activity drives the induction of trained immunity.^11^ Supporting the hypothesis of IL-38 as trained immunity-regulating factor, we show that rhIL-38 dampens mitochondrial metabolic capacity of human monocytes in a trainer-dependent fashion (Figure 1D) and regulates glycolytic activity stimulus independently (Figure 1E).

Epigenetic modifications underly the long-lived character of a trained phenotype. To link IL-38 to the induction or inhibition of trained immunity, we next assessed whether rhIL-38 affects epigenetic signatures characteristic for trained immunity. RhIL-38 consistently reduced trimethylation of histone 3 lysine 4 (H3K4me3) in promoter regions of trained immunity-associated genes (*HK2*, *PFKP*, *MTOR*, *TNF*, *IL6*, and *IL1B*)^18^^,^^32^^,^^33^ (Figure 1F). Suppressive signatures (H3K9me3) supported these observations (Data S1D). Additionally, we investigated the gene expression of four epigenetic modifiers associated with trained immunity (*SETD7*,^8^ *EHTM2*,^7^ *KDM5a*,^5^ and *KMT2E*^34^), one transposase (*SETMAR*^35^), and one lysine-methyltransferase (*PRDM9*^36^). These results identify a mechanistic avenue through which IL-38 can indirectly affect epigenetic signatures (Figure 1G).

### Endogenous IL-38 regulates trained immunity induced by BCG vaccination in humans *in vivo*

**Figure 2:**
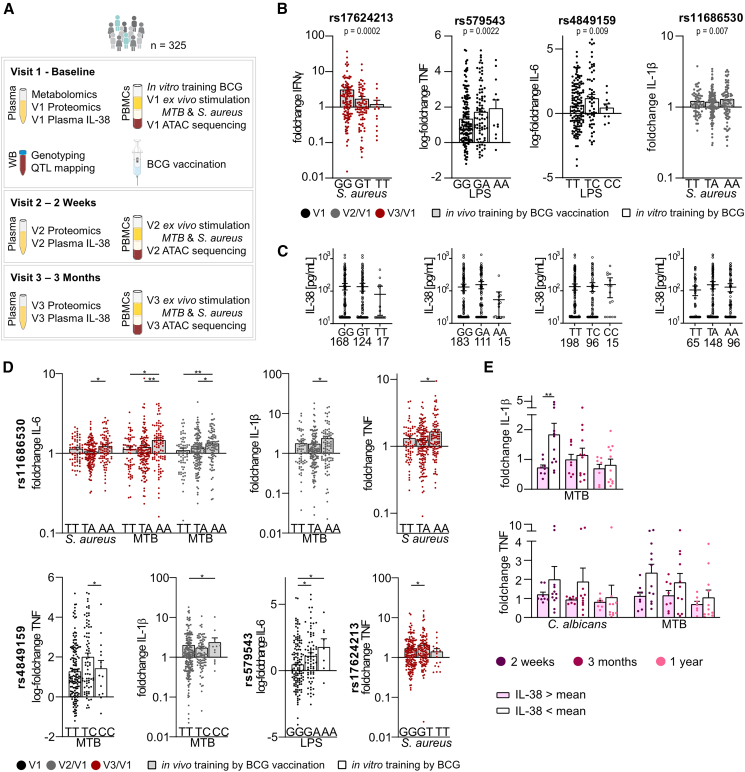
Genetic variation in *IL1F10* and circulating plasma IL-38 are linked to decreased training responses (A) Schematic overview of immunological assays performed in the 300BCG cohort. (B) SNPs rs17624213, rs579543, rs4849159, and rs1168530 mapped to *IL1F10* are associated with reduced trained immunity responses. Data are presented as mean ± SEM. (C) Genetic variation in SNPs rs17624213, rs579543, rs4849159, and rs1168530 does not affect IL-38 (pg/mL) concentrations in circulation. Data are presented as median and were analyzed by Kruskal-Wallis test. (D) Differences in cytokine responses of stimulated PBMCs upon (gray) *in vivo* induction of trained immunity by BCG vaccination and (white) *in vitro* induction of trained immunity (BCG: 5 μg/mL) are genotype dependent. Shown are fold changes of cytokine production between (black) *in vitro* trained and untrained cells, (gray) visit 1 and visit 2, or (red) visit 1 and visit 3. Cells were stimulated with LPS (10 ng/mL), *S. aureus* (10^6^ CFU/mL), or *M. tuberculosis* (5 μg/mL) for 24 h. Data are presented as mean ± SEM. Data were analyzed by Kruskal-Wallis test; ∗*p* < 0.05 and ∗∗*p* < 0.01. (E) PBMCs of 20 healthy volunteers (20BCG cohort) were stimulated for 24 h with heat-killed *M. tuberculosis* (5 μg/mL), heat-killed *C. albicans* (106/mL), or medium control at 37 °C 2 weeks, 3 months, and 1-year post-BCG vaccination. Cytokine responses of TNF and IL-1β were stratified based on circulating IL-38 plasma concentrations (cohort mean: 158.69 pg/mL). Individuals with high plasma IL-38 (pink; *n* = 7–8) had lower training responses than individuals with low plasma IL-38 (white; *n* = 12–13) IL-38. Data are shown as fold change ± SD. Data were analyzed by Kruskal-Wallis test; ∗∗*p* < 0.01.

To confirm that endogenous IL-38 regulates trained immunity *in vivo*, two existing cohorts of BCG-vaccinated, healthy adults were re-purposed. The 300BCG cohort provided data on (1) *in vitro* trained immunity responses, (2) *ex vivo* cytokine responses, (3) genotype, (4) metabolomics, (5) proteomics, and (6) Assay for Transposase-Accessible Chromatin using sequencing (ATAC-seq) from three visits (baseline [visit 1], 2 weeks [visit 2]/3 months [visit 3] post- vaccination) (Figure 2A).^33^^,^^37^^,^^38^ The 20BCG cohort included *ex vivo* cytokine responses from four visits (baseline [visit 1], 2 weeks [visit 2]/3 months [visit 3]/12 months [visit 4] post-vaccination).^39^

#### *IL1F10* polymorphisms are associated with impaired trained immunity responses

We identified four SNPs in a 100 kb range of *IL1F10* associated with trained immunity responses by QTL (quantitative trait locus) mapping (Figure 2B), one of which (rs579543) was validated in an independent cohort of 200 individuals (*p* = 0.0058) (www.humanfunctionalgenomics.org). While plasma IL-38 concentrations did not differ between genotypes (Figure 2C), differences in IL-6, IL-1β, and TNF production were observed for up to 3 months after vaccination (Figure 2D). To validate that differences in cytokine responses are indeed linked to circulating IL-38 concentrations, responses were stratified by plasma IL-38 concentrations in the 20BCG cohort (Figure 2E), showing persistently lower training in individuals with higher plasma IL-38 than in those with low plasma IL-38. This effect faded over a period of 1 year.

#### Elevated plasma IL-38 mediates a mildly anti-inflammatory milieu in circulation

**Figure 3:**
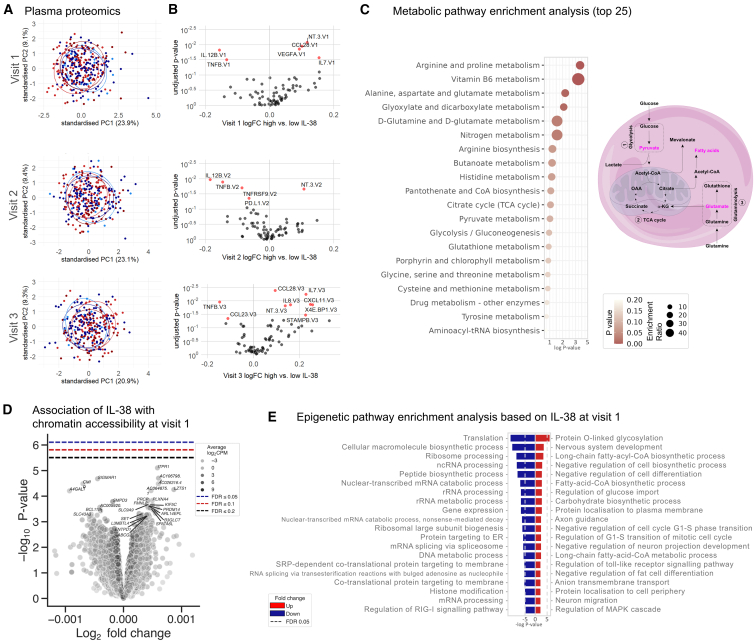
Multiomics analyses link trained immunity to circulating IL-38 (A) Principal-component analysis of 73 inflammatory markers based on circulating plasma IL-38 concentrations grouped by IQR at all three visits (300BCG cohort) shows no IL-38-dependent inflammatory pattern; visit 1 *n* = 316; visit 2 *n* = 307; visit 3 *n* = 293. (B) Volcano plots of differentially expressed proteins between individuals with high or low circulating IL-38 at visit 1 shows that IL-38 is negatively associated with inflammatory markers. Visit 1 *n* = 316; visit 2 *n* = 307; visit 3 *n* = 293. Data were analyzed by Wilcoxon matched-pairs signed rank test; ∗*p* < 0.05 (red). (C) Pathway enrichment plot based on metabolomic analyses shows the top 25 pathways of metabolites associated with IL-38 in circulation; *n* = 301. (Left) Schematic overview of the metabolic pathways affected by IL-38. Metabolites with negative correlation to IL-38 are shown in pink. (D) Chromatin accessibility, represented as scatterplots of (−log10) *p* values derived from comparing differential accessibility by Wilcoxon matched-pairs signed rank test, does not differ between individuals with high and low IL-38 at visit 1; *n* = 301. (E) Pathway enrichment analysis of the top 1,000 scoring regions of chromatin accessibility associated with IL-38 in circulation at visit 1 based on the GO (Gene Ontology) Biological Processes 2018 library. See also Data S2 and Data S3.

To explore systemic effects of IL-38 on immune responses, we analyzed 73 circulating markers of inflammation in the 300BCG cohort. We found no association between expression patterns and baseline IL-38 in a principal-component analysis (PCA) (Figure 3A). Yet, some markers were differentially expressed in a direct comparison of individuals with high and those with low plasma IL-38 (Figure 3B; logFC corresponds to differences of |7–15|%), which was reflected in a correlation analysis (Table 1). We observed mild negative associations between IL-38 and the expression of mainly pro-inflammatory markers (IL-7, CCL20/25/28, CXCL1/6/11, IL-8, and CCL13). Changes in these associations directly after BCG vaccination were transient and reverted to baseline 3 months after the vaccination. These data show that circulating IL-38 dampens acute responses upon vaccination. Whether these relatively small differences are of biological relevance remains to be experimentally and clinically validated.

**Table 1:** Proteomics—Relation of IL-38 with circulating markers of inflammation

Visit	Gene ID	Spearman^a^		Wilcoxon^b^		Visit	Gene ID	Spearman		Wilcoxon	
		*p* value	R value	*p* value	FC			*p* value	R value	*p* value	FC
1	*IL18R1*	0.0302	−0.1307	–	–	3	*CXCL1*	0.0153	−0.1543	–	–
1	*TNFB*	0.0210	0.1390	0.0314	−0.9114	3	*CXCL11*	0.0048	−0.1781	0.0135	0.1872
1	*IL12B*	–	–	0.0151	−0.8973	3	*CXCL6*	0.0206	−0.1477	–	–
1	*VEGFA*	0.0144	−0.1470	0.0142	0.0664	3	*GDNF*	0.0197	−0.1487	–	–
1	*NT3*	0.0249	−0.1352	0.0086	0.0843	3	*HGF*	0.0246	−0.1436	–	–
1	*CCL28*	0.0412	−0.1234	0.0114	0.0725	3	*IL8*	0.0037	−0.1833	0.014	0.1178
1	*IL7*	–	–	0.0276	0.1129	3	*TGFB1*	0.0042	−0.1810	–	–
2	*NT3*	0.0441	−0.1242	0.0219	0.0768	3	*MCP4*	0.0095	−0.1645	–	–
2	*IL12B*	–	–	0.0107	−0.8798	3	*NT3*	0.0129	−0.1580	0.0153	0.1004
2	*TNFB*	–	–	0.0128	−0.9038	3	*VEGFA*	0.0372	−0.1336	–	–
2	*TNFRSF9*	–	–	0.0199	−0.9422	3	*IL7*	–	–	0.0057	0.1715
2	*PDL1*	–	–	0.0441	−0.9567	3	*EIF4EBP1*	–	–	0.0139	0.1949
3	*CCL20*	0.0391	−0.1324	–	–	3	*STAMPB*	–	–	0.0339	0.1703
3	*CCL25*	0.0488	−0.1270	–	–	3	*TNFB*	–	–	0.011	−0.9031
3	*CCL28*	0.0027	−0.1889	0.0041	0.0679	3	*CCL23*	–	–	0.045	−0.9258

a Circulating plasma IL-38 concentrations were used as continuous variable to calculate correlations.

b The mean value of circulating plasma IL-38 concentrations was used as cutoff grouping value.

#### Metabolic activity inversely correlates with circulating IL-38 concentrations

We next investigated the impact of circulating IL-38 on immunometabolism, identifying 58 metabolites correlated with IL-38 (Data S2), and an additional ten that differed between individuals with high or low plasma IL-38 (see Data S3 for fold changes). Pyruvate and glutamate, mapped to glutathione and pyruvate metabolism, glycolysis, and the Krebs cycle, were negatively correlated to IL-38 (Figure 3C), all of which are linked to the induction of trained immunity.^33^ Further, we identified inverse correlations between IL-38 and fatty acids, phospholipids, and the fatty acid derivative propylhydroxypentanoic acid—all substrates of lipid metabolisms. As also the synthesis of lipid mediators is crucial for trained immunity responses of human monocytes,^40^ these data together strengthen the hypothesis that IL-38 regulates trained immunity by targeting multiple metabolic pathways involved in its induction.^33^^,^^40^

#### Circulating IL-38 affects chromatin accessibility of epigenetic modifiers

Based on our *in vitro* observations, we hypothesized that IL-38 shapes epigenetic signatures *in vivo*. Studying chromatin accessibility relative to IL-38 (visit 1), we identified weak positive associations between IL-38 and the epigenetic modifiers *PRDM14*, *SET*, and *L3MBTL4* (Figure 3D), which was reflected in a pathway enrichment analysis (histone modifications) (Figure 3E). Additional gene hits included in this pathway are *HDAC1* (Histone Deacetylase 1); *SIRT2* (deacetylases Sirtuin 2); *KDM4A* (Lysine Demethylase 4A), *PRMT1* and *PRMT2* (Protein Arginine Methyltransferase 1 and 2); transcriptional coactivators *EP300* (E1A Binding Protein P300) and *EYA3* (EYA Transcriptional Coactivator and Phosphatase 3); and transcriptional regulators *SIN3A* (SIN3 Transcription Regulator Family Member A), *RUVBL2* (RuvB Like AAA ATPase 2), *MORF4L1* (Mortality Factor 4 Like 1), and *ATXN7* (Ataxin 7). Taken together with the experimental *in vitro* data, these observations suggest that IL-38 affects the expression epigenetic modifiers.

### IL-38 dampens trained immunity-mediated infection control

#### Endogenous IL-38 inhibits trained immunity in murine bone marrow *in vivo*

**Figure 4:**
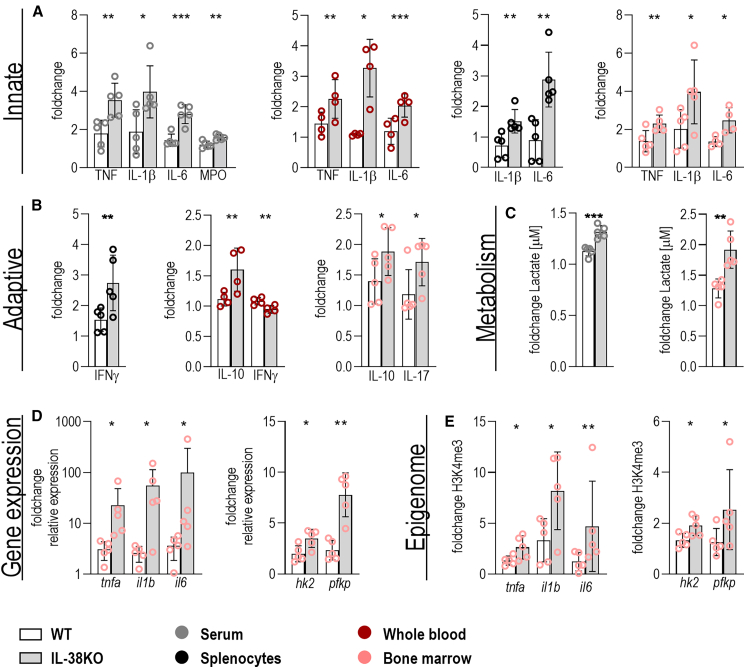
Induction of trained immunity is higher in IL-38KO than WT mice WT (white) and IL-38KO (gray) mice were trained i.p. with β-glucan (1 mg/mouse) for 7 days followed by LPS (1 mg/mouse) injection and sacrifice after 4 h. Cytokine and lactate production were assessed in serum (gray) and in cell culture supernatants of bone marrow (rose), whole blood (red), and splenocytes (black). Training responses were assessed by production of (A) IL-1β, IL-6, TNF, and MPO; (B) IFNγ, IL-10, and IL-17; (C) lactate; and (D) transcriptomic and (E) epigenomic analyses of *tnfa*, *il1b*, *il6*, *hk2*, and *pfkp*. Trained immunity was higher in KO animals compared to WT animals; bone marrow, serum, splenocytes: *n* = 5; whole blood: *n* = 4. Data are shown as mean ± SD and analyzed by one-tailed, unpaired *t* test, ∗*p* < 0.05, ∗∗*p* < 0.01, ∗∗∗*p* < 0.001. See also Data S4.

To further strengthen the observations that IL-38 regulates trained immunity *in vivo*, we next investigated trained immunity signatures in serum, splenocytes, whole blood, and myeloid progenitors in mice. IL-38KO and WT mice were trained with β-glucan and challenged with LPS for 4 h (Figure 4 and Data S4A). We observed consistently higher training responses for IL-1β and IL-6 and partly for TNF, myeloperoxidase (MPO), interferon (IFN)γ, IL-10, and IL-17 in IL-38KO over WT in serum and cultures of splenocytes, whole blood, and bone marrow (Figures 4A and 4B). Baseline responses of IL-38KO mice were equal or lower than in WT animals (Data S4B). Also lactate production was higher in the IL-38KO model than in WT animals (Figure 4C). Gene expression profiles and epigenetic signatures mirrored protein measurements of cytokines and lactate in cell culture supernatants (Figures 4D and 4E), collectively showing a role for IL-38 in the induction of trained immunity. Importantly, we demonstrate an inhibitory effect of endogenous IL-38 not only on circulating cells but also on bone marrow level.

#### Trained immunity-conferred protection against infections is impaired by IL-38 *in vitro* and *in vivo*

**Figure 5:**
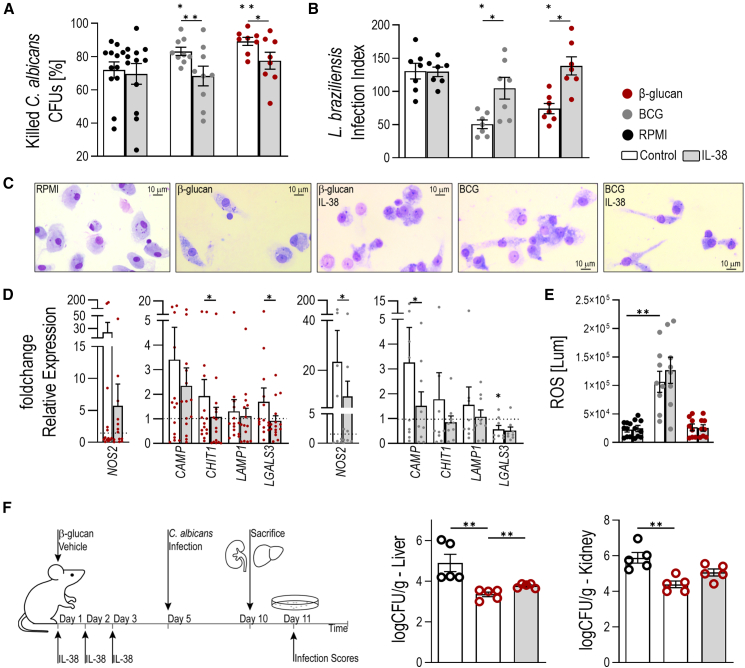
Trained immunity-associated microbicidal activity is decreased by rhIL-38 Human monocytes were infected (A) with *C. albicans*, *n* = 8, or (B) with *L. braziliensis*, *n* = 7, for 24 h. Trained cells (β-glucan [1 μg/mL; red], BCG [5 μg/mL; gray]) show a higher killing capacity than untrained cells (black), which was opposed by pre-incubation with rhIL-38 (90 ng/mL; grey-filled). Data are shown as ± SEM. (C) Infection index was evaluated light-microscopically after Giemsa staining with a 40× magnification; *n* = 7. (D) *In vitro*-trained human monocytes (pre-incubated with rhIL-38 [90 ng/mL; grey-filled]); trained with β-glucan [1 μg/mL; red] or BCG [5 μg/mL; gray]) have increased expression of microbicidal molecules. RhIL-38 opposes this effect; *n* = 8. Data are presented as mean ± SD. (E) BCG (5 μg/mL; gray) but not β-glucan (1 μg/mL; red) increases ROS production in human monocytes; *n* = 8. Data are presented as mean ± SEM. (F) Schematic overview of the murine *in vivo* infection model (left). Fungal burden in the liver (middle) and spleen (right) upon *C. albicans* infection in the ocular pouch is reduced by β-glucan training (1 mg/mouse), which is reversed by rhIL-38 (1 μg/mouse/day); *n* = 5. Data are presented as mean ± SEM. Data were analyzed by (A–E) two-tailed Wilcoxon matched-pairs signed rank test; ∗*p* < 0.05 and ∗∗*p* < 0.01 or (F) two-tailed, unpaired *t* test; ∗*p* < 0.05. Asterisks in the top row represent results comparing training conditions to the RPMI control. Asterisks in the bottom row compare IL-38 and control condition within each training condition. See also Data S5.

As trained immunity reduces infectious burden *in vitro*^41^ and *in vivo*,^15^^,^^16^^,^^17^^,^^42^^,^^43^ we next investigated whether rhIL-38 impairs trained immunity-driven microbicidal activity. Eradication of *L. braziliensis* and *C. albicans* by trained human monocytes was more efficient than in naive cells and those cells trained in the presence of rhIL-38 (Figures 5A and 5B and Data S5). A prominent vacuolar system and elongated shape indicates higher phagocytic activity and motility^44^ of trained cells infected with *L. braziliensis*. While vacuolization appears reduced by rhIL-38, a difference in elongation was not observed (Figure 5C), indicating an effect on phagocytosis in line with previous findings.^45^ Further, we observed an overall increase of the leishmanicidal and anti-fungal proteins iNOS (*NOS2*),^46^ cathelicidin (*CAMP*),^41^^,^^47^^,^^48^ chitotriosidase 1 (*CHIT1*),^49^ lysosomal-associated membrane protein 1 (*LAMP1*),^50^ and partly of galectin 3 (*LGALS3*)^51^ in trained cells (Figure 5D). The expression of all molecules was downregulated by rhIL-38. Reactive oxygen species (ROS) production^41^ was not affected by rhIL-38 (Figure 5E).

These findings were corroborated by a murine *in vivo* infection model with *C. albicans* (Figure 5F). Fungal burden was significantly lower in organs of trained mice, which was impaired by IL-38. These results underline that variations in circulating IL-38 may affect the susceptibility to infections.

## Discussion

Trained immunity is well studied for its protective properties in infection clearance and is being explored as a tool for anti-cancer therapy.^15^^,^^16^^,^^42^ Its contribution to pathophysiological inflammation and associated treatment avenues, however, remain underappreciated. We propose that IL-38 acts as endogenous inhibitor of trained immunity, which we investigate here using two cohorts of healthy adults. *In vitro*, we explore underlying molecular pathways in circulating cells and myeloid progenitors and elucidate functional consequences in the context of infections, which we validate in murine *in vivo* and human *in vitro* models. Our findings show that IL-38 acts as endogenous inhibitor of trained immunity in humans *in vivo*, which impairs trained immunity-mediated infection control. Importantly, we demonstrate that IL-38 inhibits the induction of bone marrow-mediated trained immunity.

We show *in vitro* that IL-38 dampens trained immunity by in humans in a training stimulus-independent fashion. Reduced secondary cytokine responses, mediated by rhIL-38, are accompanied by lower metabolic activity and epigenetic changes. We further provide evidence that IL-38 impairs protective training responses against fungal and parasitic infections, which we substantiate in a murine *in vivo* infection model. This observation is clinically relevant, as individuals with high circulating IL-38 may experience more (opportunistic) infections.

Human *in vitro* data are validated in murine bone marrow, demonstrating that IL-38 also affects bone marrow-mediated trained immunity, which is prerequisite for innate immune memory as a long-lasting phenotype. In trained mice, the frequency and number of hematopoietic progenitor cells (Lin−cKit+Sca1+) and multipotent progenitors (MPPs; CD48^+^CD150−LSK) are higher than those in naive mice, which is associated with an increase in the frequency of the myeloid-biased subset at cost of the lymphoid-biased subset.^52^^,^^53^ Within the myeloid progenitors (Lin−cKit+Sca1−), trained immunity changes the ratio between granulocyte-monocyte progenitors (GMPs) and common myeloid progenitors, favoring GMPs.^53^ BCG vaccination in humans elicits trained immunity in a similar fashion via the hematopoietic progenitor compartment, introducing a persistent myeloid bias.^54^ We observe training responses in the IL-38KO animals that can be assigned to a higher GMP and MPP frequency (MPO, IL-6, TNF, and IL-1β). Precise effects of IL-38 on trained immunity-induced changes in the bone marrow niche remain to be investigated.

Epigenetic signatures, namely an increase of H3K27ac, H3K4me3, and H3K4me1 accompanied by a decrease of H3K9me2 and H3K9me3, are associated with a trained phenotype.^25^ ATAC-seq data suggest that IL-38 regulates epigenetic modifiers, which is supported by experimental *in vitro* data. The methyltransferase *SETD7*, previously linked to the induction of trained immunity,^6^ is downregulated by rhIL-38. Additionally, a lysine-demethylase (*KDM5a*) and a lysine-methyltransferase (*EHTM2*), both of which repress trained immunity induction,^5^^,^^7^ were upregulated by rhIL-38. Whether altered expression of these enzymes by IL-38 regulates a trained phenotype *in vivo* remains to be investigated.

In a cohort of 325 healthy adults, we find four genetic variants in *IL1F10* (chr2: 113,825,547-113,833,427) linked to lower training responses, one of which was validated in an independent cohort (rs579543). These findings corroborate previous study results (rs58965312).^28^ It would be valuable to evaluate whether rs58965312 affects IL-38 plasma concentrations, especially as this was not the case for the here identified variants. Importantly, future studies should employ plasma protein detection techniques that can detect and ideally distinguish between multiple IL-38 truncation variants, as not all known variants have the same biological activity.^55^ It also has to be noted that the effect on trained immunity may not be attributed to variation in circulating IL-38 concentrations but to the variant’s proximity to other genes involved in induction and regulation of trained immunity. Rs11686530 (chr2:113770464) lies between *IL36A* and *IL36B*, which have been linked to inducing trained immunity.^14^ Similarly, rs579543 (chr2:113889631) lies in *IL1RN*, encoding for an inhibitor of trained immunity.^25^ Rs17624213 (chr2:113933247) and rs4849159 (chr2:113925061) are not associated with trained immunity thus far. Validation of these SNPs in the context of IL-38-mediated regulation of trained immunity is therefore required.

Proteomic analyses underline the anti-inflammatory character of IL-38 but highlight only one association (IL-38 and 4) as potentially trained immunity specific. Importantly, the observed IL-38-associated increase in the expression of inflammatory markers was overall small, which is in line with IL-38 not being an acute phase cytokine.^56^ Yet, its expression is dysregulated in ample chronic inflammatory conditions^57^ and shows a wide range of plasma concentrations in healthy adults,^58^ the reasons as to why are thus far not known. One can speculate that persistently elevated expression of inflammatory proteins associated with reduced expression of IL-38 may accumulate and show systemic pathological effects. We hypothesize in this context that IL-38 may qualify as second-line treatment for pathological inflammation or may serve as a biomarker for persistent, low-grade, systemic inflammation.

Based on the presented data, we conclude that IL-38 regulates the induction of trained immunity in humans after vaccination with BCG. We show regulation of all hallmarks of trained immunity with functional consequences on infection control. We propose that IL-38-targeting drugs may boost antimicrobial responses in individuals with high circulating IL-38. Moreover, we propose IL-38 as second-line treatment for auto-inflammatory patients who are treatment refractory to standard of care. Murine models of chronic inflammation are warranted to further explore the therapeutic potential.

### Limitations of this study

For the *in vitro* studies, data on sex were not collected, and the murine studies have only been performed in male mice; this limits the generalizability of this study. Furthermore, it is important to notice that the anti-inflammatory bioactivity of IL-38 varies with N-terminal processing^58^ and that not all described isoforms are anti-inflammatory (full-length, 3-152aa, 5-152aa, and 20-152aa).^55^ A recent study shows that keratinocyte-derived IL-38 undergoes methionine excision and starts at cysteine 2 (2-152aa).^31^ As commercial IL-38 ELISA kits detect multiple of these variants, we cannot conclusively determine which variant(s) were detected in the here analyzed cohorts. This technical limitation may have convoluted the observed associations with trained immunity-related pathways and responses as well as the effect of identified SNPs on biologically active variants of IL-38. Based on *in vitro* data, we can, however, state that rh3-152aa regulates trained immunity. *In vitro* validation of the regulatory characteristics on trained immunity of other truncation variants is warranted.

## Resource availability

### Lead contact

Requests for further information and resources should be directed to and will be fulfilled by the lead contact, Dr. Rob J.W. Arts (rob.jw.arts@radboudumc.nl).

### Materials availability

This study did not generate new unique reagents.

### Data and code availability

This paper analyzes existing, publicly available data, accessible from the original papers as mentioned in the STAR Methods section. This paper does not report original code. Any additional information required to reanalyze the data reported in this paper is available from the lead contact upon request. All used data of the “300BCG” cohort^33^^,^^37^^,^^38^^,^^39^^,^^59^ and the “20BCG” cohort^39^ are available in the original publications. Additionally measured IL-38 plasma concentrations are available upon request from the corresponding author.

## Acknowledgments

The authors would like to thank all participating patients and volunteers of the 300BCG and the 20BCG cohorts for their participation. We further thank Profa. Dra. Fátima Ribeiro-Dias (Laboratório de Imunidade Natural [LIN], Instituto de Patologia Tropical e Saúde Pública, Universidade Federal de Goiás, Goiânia, Goiás, Brazil) for kindly providing the *Leishmania* spp. and Christoph Bock (Medical University of Vienna, Institute of Artificial Intelligence, Center for Medical Data Science, Austria & CeMM Research Center for Molecular Medicine of the Austrian Academy of Sciences, Vienna, Austria) for his support in writing the manuscript. R.J.W.A. is supported by the VENI grant (09150161810007). M.G.N. is supported by an Advanced grant of the 10.13039/100010663European Research Council (ERC, #833247). L.F. was supported by a 10.13039/100010665Marie Skłodowska-Curie Actions Individual Fellowship (grant agreement no. 842480). R.t.H. was supported by an 10.13039/100004410EMBO Postdoctoral Fellowship (grant agreement no. ALTF 702–2020). C.D. is supported by the 10.13039/100000002NIH grant AI15614.

## Author contributions

Conceptualization: L.U.T., R.J.W.A., and L.A.B.J.; methodology: L.U.T., S.J.C.F.M.M., T.K., J.C.d.S., C.M.M.-M., D.M.d.G., R.J.W.A., and L.A.B.J.; formal analysis: L.U.T., V.M., L.F., and R.t.H.; writing – original draft: L.U.T.; writing – review and editing: L.U.T., V.M., R.t.H., T.K., M.G.N., D.M.d.G., R.J.W.A., and L.A.B.J.; validation: L.U.T.; visualization: L.U.T.; investigation: L.U.T.; data curation: L.U.T., V.M., L.F., and R.t.H.; funding acquisition: C.D., M.G.N., and R.J.W.A.; resources: M.G.N., R.J.W.A., and L.A.B.J.; supervision: R.J.W.A. and L.A.B.J.; project administration: R.J.W.A. and L.A.B.J.

## Declaration of interests

M.G.N. and L.A.B.J. are scientific founders of Trained Therapeutix and Discovery (TTxD). M.G.N. is a scientific founder of Biotrip.

## STAR★Methods

### Key resources table

**Table:** 

REAGENT or RESOURCE	SOURCE	IDENTIFIER
**Chemicals, peptides, and recombinant proteins**		

rhIL-38 (3-152aa)	Professor Charles Dinarello, University of Denver, Colorado, USA	N/A
IL-1Ra (Anakinra)	MedChemExpress	Cat# 143090-92-0
IL-1α	R&D Systems	Cat# 200-LA-002/CF
β-glucan (β 1,3-(D)-glucan	Professor David Williams, College of Medicine, Johnson City, USA	N/A
Lipopolysaccharide	Sigma-Aldrich	Cat#L2880; *E. coli* serotype 055:B5
Grace’s insect medium	Gibco, Life Technologies	Cat#11595-030
Heat-inactivated fetal bovine serum (FBS)	Gibco, Life Technologies	Cat#10500064
Giemsa	Merck Millipore	Cat#1.09204.0100
Percoll	Sigma-Aldrich	Cat#P1644
Ficoll-Paque	GE Healthcare	Cat#17-1440-03
Roswell Park Memorial Institute medium (RPMI)	Invitrogen	Cat#22406031
16% Formaldehyde	Fisher Scientific	Cat#28908, 11835835
Zymosan	Sigma-Aldrich	Cat#Z4250
2-deoxy-D-glucose	Merck	Cat#D6134
Oligomycin A	Merck	Cat#75351-5 MG
FCCP	Merck	Cat#C2920-10 mg
Antimycin	Merck	Cat#A8674-25 mg
Rotenone	Merck	Cat#R8875-1G
Luminol (5-amino-2,3, dihydro-1,4-phtalazinedione)	Merck	Cat#521-31-3
SYBR Green	Applied Biosciences	Cat#4368708
Bacille Calmette-Guérin Vaccine	Intervax, Canada/AJ vaccines, Denmark	BCG-Bulgaria strain/Danish 1331 sub-strain
Bovine Serum Albumin (BSA)	Sigma-Aldrich	Cat#A7030
Protein A/G PLUS-Agarose	Santa Cruz Biotechnology	Cat# sc-2003
Glycine	Merck	Cat# 1042010100

**Critical commercial assays**		

Human/Murine TNF ELISA	R&D Systems	Cat#DY210/MTA00B-1
Human/Murine IL-6 ELISA	R&D Systems	Cat#DY206/DY406-05
Human IL-38 ELISA	R&D Systems	Cat#DY9110-05
Human/Murine IL-IFNγ ELISA	R&D Systems	Cat#DY285B-05/DY483-05
Human/Murine IL-1 β ELISA	R&D Systems	Cat#DY201/
Murine IL-10 ELISA	R&D Systems	Cat#DY417-05
Murine MPO ELISA	R&D Systems	Cat#DY3667
iScript cDNA Synthesis Kit	Bio-Rad	Cat#1708891
RNeasy Mini Kit	Quiagen	Cat#74104
Lactate Fluorometric Assay kit	Biovision	Cat#K607
Inflammation panel	OLINK proteomics	N/A

**Deposited data**		

Human functional genomics project		300BCG

**Experimental models: Organisms/strains**		

*Candida albicans*	N/A	ATCC MYA-3573, UC820
*Leishmania Viannia braziliensis*	Leisbank IPTSP/UFG	MHOM/BR/2003/IMG
C57BL/6	Jackson Laboratory	N/A
C57BL/6	The et al.^60^	IL-38KO

**Oligonucleotides**		

Primers for qPCR, see Tables 3 and 4	This paper	N/A
Primers for ChIP-PCR, see Tables 2 and 4	This paper	N/A

**Software and algorithms**		

GraphPad Prism	Graphpad Software	https://www.graphpad.com
R statistical programming	N/A	RRID: SCR_001905

### Experimental model and study participant details

#### *In vivo* model of trained immunity in mice

Male C57BL/6 (Jackson Laboratory (Bar Harbor, USA)) and IL-38 knock out (KO) mice^60^ were randomly assigned to groups (*n* = 5). The murine trained immunity and infection models were performed independently with 5 animals per experimental group, respectively. Experiments followed the Institutional Animal care and Use Committees (University of Colorado, Denver, ethical approval number protocol #00105).

#### *In vitro* model of trained immunity in humans

Buffy coats were obtained from Sanquin Blood Bank, Nijmegen, The Netherlands. Additionally, blood was collected in EDTA from healthy volunteers after obtaining written informed consent. The study was approved by Medical Ethical Committee Oost-Nedederland (NL32357.091.10). Information on sex was not collected.

#### *In vivo* model of trained immunity in humans

Studies were approved by the ethical committee of the Radboud University and Radboudumc Nijmegen and Arnhem-Nijmegen Medical Ethical Committee (300BCG: NL58553.091.16; 20BCG: NL32357.091.10) and performed according to the Declaration of Helsinki. Volunteers gave written informed consent. Cohorts “300BCG” (www.humanfunctionalgenomics.org) and “20BCG” consist of 325 (57% female, 18–71 years)^33^^,^^37^ and 20 (information on sex was not collected; 20–36 years)^39^ healthy adults, respectively. *In vitro* and *in vivo* models are described elsewhere in detail.^38^^,^^39^ Multi-omics analyses have been described elsewhere.^33^^,^^37^^,^^38^^,^^39^^,^^59^ Details on the re-analysis are described below.

### Method details

#### *In vitro* model of trained immunity for human monocytes

##### Cell isolation and induction of trained immunity

Peripheral blood mononuclear cell (PBMC) were isolated from buffy coats (Sanquin Blood Bank, Nijmegen, The Netherlands) or venous blood collected in EDTA from healthy volunteers who gave informed consent by differential density centrifugation over Ficoll-Paque (GE healthcare, UK). Cells were quantified (XN-45 haematology analyser; Sysmex Corporation, Japan). Isolation of monocytes was performed as described elsewhere.^61^ Cells were cultured in Dutch modified RPMI 1640 culture medium (Invitrogen, CA, USA) supplemented with 5 μg/mL gentamicin, 2 mM glutamax (GIBCO, Life Technologies, CA, USA), and 1 mM pyruvate (GIBCO).

For chromatin precipitation and seahorse assays, 15 × 10^6^ cells/5 mL per dish were adhered on polystyrene petri dishes with a 10 cm diameter (Corning, NY, USA) for 1 h at 37 °C, 5% CO_2_. Non-adherent cells were washed out with warm PBS. For assessment of reactive oxygen species (ROS) and killing assays, monocytes were cultured in sterile FACs tubes (15 × 10^6^ cells/3 mL per tube). For cytokine and lactate measurements as well as for qPCR, PBMCs were plated in supplemented RPMI at a density of 5 × 10^5^ cells/well in flat-bottom 96-well plates (Corning), allowed to adhere for 1 h at 37 °C, 5% CO_2_, and non-adherent cells were washed out in three steps with warm PBS.

Trained immunity was induced *in vitro* as described previously with minor modifications.^32^ Monocytes were obtained as described above and pre-incubated with recombinant human (rh) IL-38 (3-152aa) for 1 h at 37 °C, 5% CO_2_ (25 or 90 ng/mL depending on the batch; Data S1A; Elan Eisenmesser (University of Colorado, Denver, CO, USA)) or supplemented RPMI containing 10% human pooled serum, referred to as complete medium, as negative control. Training was induced by Bacillus Calmette-Guerin (BCG) (5 μg/mL; Danish 1331 sub-strain; AJ vaccines, Denmark), β-1,3-(D)-Glucan (5 μg/mL; Professor David Willems (College of Medicine, Johnson City, TN, USA)), or IMG3 lysates (25 μg/mL; strain MHOM/BR/2003/IMG). After for 24 h at 37 °C, 5% CO2, cells were washed with warm PBS and incubated in complete medium for five days at 37 °C, 5% CO2 with one medium change.

On day six, cells trained on polystyrene petri dishes were harvested using Versene (GIBCO) for 30 min at 37°C. 1.5 × 10^6^ cells/condition were collected for epigenetic assays. Remaining macrophages were replated in quadruplicates in supplemented RPMI on Seahorse cell-culture 96-well plates (10^5^ cells/well) for metabolic assays. Cells trained in FACs tubes were harvested and plated in supplemented RPMI in triplicates on white flat-bottom 96-well plates (2 × 10^5^ cells/well) for ROS measurements, on flat-bottom 96-well plates (10^5^ cells/well) for killing assays with *Candida albicans*, and on aseptic 12 mm coverslips (2 × 10^5^ cells/well) for killing assays with *Leishmania braziliensis*. Cells trained in 96-well plates were re-stimulated with lipopolysaccharide (LPS) (1 ng/mL; derived from *E. coli* serotype O55:B5; Sigma-Aldrich) in complete medium for 24 h at 37 °C, 5% CO2, and supernatants were stored at −20°C until further analysis. Alternatively, cells were collected in RLT buffer (Qiagen GmbH, Germany) in duplicates/condition and snap-frozen until RNA isolation.

##### Epigenetic analyses (ChIP-qPCR)

DNA (of approximately 10^6^ cells/mL) was cross-linked in methanol-free 1% formaldehyde (Sigma-Aldrich) and 1.25 M of glycine. Fixed cells were stored in PBS at 4 °C before sonication in ten cycles (30 s on/30 s off) using a Diagenode Bioruptor. Supernatant containing chromatin was stored at −80°C.

For immunoprecipitation, beads (Protein A/G PLUS-Agarose; Santa Cruz Biotechnology, USA) were prepared as indicated by the manufacturer and incubated overnight at 4 °C with 60% of chromatin with H3K4me3 (1.3 μg; Diagenode Diagnostics, Belgium) or H3K9me3 (1 μg; Diagenode) antibodies. The remainder was used as total input. Chromatin washed and eluted, and DNA was purified over MiniElute DNA purification kits (Qiagen GmbH, Hilden, Germany). RT-qPCR was performed to determine the percentage of H3K4me3-and H3K9me3-positive DNA. Input samples were diluted 25x and ChIP samples 3x in H_2_O. Samples were analyzed by comparative Ct-method on the StepOne PLUS qPCR machine (Applied Biosystems, CA, USA) using SYBR green (Invitrogen). Primers used are listed in Table 2. GADPH and MB were used as negative control, and ZNF UTR and GADPH were used as positive control for H3K9me3 and H3K4me3, respectively.

**Table 2:** Epigenetic primers used for ChIP-PCR

Gene	FW	RV
*TNF*	CAGGCAGGTTCTCTTCCTCT	GCTTTCAGTGCTCATGGTGT
*MTOR*	ATAAAGAGCGCTAGCCCGAA	GACCCCTCCCGGTGTAATTC
*IL6*	AGGGAGAGCCAGAACACAGA	GAGTTTCCTCTGACTCCATCG
*IL1B*	CATGGCTGCTTCAGACACCT	ACACATGAACGTAGCCGTCA
*IL1RA*	TTGCGACACTTAGTGGGGTT	CGGAAATACCCTCCTCGCAT
*PFKP*	CGAAGGCGATGGGGTGAC	ATCTTGCGGGCCACTAGAAG
*HK2*	GAGCTCAATTCTGTGTGGAGT	ACTTCTTGAGAACTATGTACCCTT
*GAPDH*^a^	CCCCGGTTTCTATAAATTGAGC	AAGAAGATGCGGCTGACTGT
*MB (Myoglobin)*^a^	AGCATGGTGCCACTGTGCT	GGCTTAATCTCTGCCTCATGAT
*ZNF UTR*^a^	AAGCACTTTGACAACCGTGA	GGAGGAATTTTGTGGAGCAA

a GADPH and MB were used as negative, and ZNF UTR and GADPH as positive control for H3K9me3 and H3K4me3, respectively.

##### Metabolic analysis - Extracellular flux measurements

Real-time oxygen consumption rate (OCR) was analyzed using an XF-96 Extracellular Flux Analyzer (Seahorse Bioscience, Agilent, CA, USA). Percoll monocytes were adhered for 1 h at 37 °C, 5% CO_2_, and non-adherent cells were removed together with the medium. Cells were incubated for 1 h at 37 °C (non-CO_2_-corrected) in unbuffered Seahorse medium (8.3 g DMEM powder, 0.016 g phenol red, and 1.85 g NaCl/1 L H_2_O; sterile filtered; pH 7.4 at 37 °C) containing glucose (11 mM), L-glutamine (2 mM) (Sigma-Aldrich), and pyruvate (1 mM). The assay was performed in a calibrated sensor cartridge (Seahorse Bioscience). Basal metabolic rates were determined during three consecutive measurements in Seahorse medium. OCR was determined over three measurement cycles upon injection of oligomycin (1 μM), FCCP (1 μM), rotenone/antimycin A (1.25/2.5 μM) (Sigma-Aldrich).

##### Reactive oxygen species (ROS) quantification

A luminol-enhanced luminescence assay was used to determine the production of ROS. Luminol (10^−3^ M in HBSS, 0.5% BSA), trained monocytes (2 × 10^5^ cells/well), and serum-opsonised zymosan (0.8 μg/mL) were plated on white flat-bottom 96-well plates (Corning) in a 1:10:1 ratio. Opsonised zymosan was prepared by incubation of *Saccharomyces cerevisiae* zymosan (Sigma-Aldrich) in fresh human serum for 30 min at 37 °C, after which the particles were washed twice in PBS and suspended in PBS. Chemiluminescence was measured every 142 s for 1 h.

#### Killing assays

##### Candida albicans

Live *Candida albicans* (strain *ATCC MYA-3573,* UC820) yeast was grown overnight in Sabouraud liquid medium (Thermo Scientific, CA, USA) at 30 °C, 140 rpm. Cells were centrifuged, washed three times with PBS, and adjusted to 1 × 10^8^ yeast cells/mL. Multiplicity of infection (MOI) of 2:1 was used for 24 h at 37 °C, 5% CO_2_ in a total volume of 0.2 mL complete medium to infect macrophages. Medium and washing fractions of four washes (0.2 mL ddH_2_O/well/wash) were combined, and 10× diluted suspension (0.05 mL/0.5 plate) was plated on Sabouraud glucose agar plates (Becton Dickinson GmbH, Germany). *C. albicans* colonies were grown for 24 h at 37 °C, 5 %CO_2_ before counting colony forming units (CFUs).

##### Leishmania braziliensis

*L. braziliensis* (strain MHOM/BR/2003/IMG) was obtained as described elsewhere.^62^ Pro-cyclic promastigotes were cultured in Grace’s insect medium (GIBCO) supplemented with 20% heat-inactivated fetal bovine serum (GIBCO), 100 U/mL penicillin/streptomycin (Sigma-Aldrich), and 10 mM GlutaMAX at 26 °C in flat-bottom 24-well plates. Parasite growth was assessed every two days, the medium was refreshed, and the culture was expanded for maintenance and metacyclogenesis. Stationary-phase parasites (metacyclic promastigotes) were obtained on day six of growth without medium refreshment. Parasites were washed three times with PBS (1000 × g, 10 min, 10 °C) and suspended in 1 mL complete medium for quantification by hemocytometer after fixation with PBS/0.4% paraformaldehyde (Thermo Scientific). MOI of 5:1 was used for 24 h at 37 °C, 5 %CO_2_ in 0.5 mL complete medium for infection. Medium was discarded, coverslips were washed once with warm PBS, and cells were fixed for 1 min in methanol. Coverslips were dried overnight, stained with Giemsa (20× in ddH_2_O) for 5 min, and mounted on glass slides once dried completely. Percentage of infected macrophages of 100 totally counted cells and number of parasites per infected cell were microscopically evaluated. Infection index was calculated as the percentage of infected cells × mean amastigotes/cell.

#### Lactate assay

Lactate concentrations were measured in cell culture supernatants on day 7 of the training protocol using a lactate fluorometric assay using Na-L-Lactate (Sigma Aldrich), Lactate oxidase (Sigma Aldrich), HRP (Thermo Scientific), and Amplex Red (Life Technologies, The Netherlands). Prior, lactate dehydrogenase was denatured by perchloric acid (PCA) precipitation using 13.7% PCA and 4 N NaOH for neutralisation. Supplemented RPMI was used as negative control.

#### Quantitative RT-PCR

RNA was isolated on day 6 of the training protocol with RNeasy column isolation kits according to manufacturer’s instructions. RNA was eluted in RNase-free water, and cDNA was synthesised by reverse transcription with iScript (Bio-Rad, CA, USA). Diluted cDNA was used for qPCR performed by the StepOne PLUS sequence detection system (Applied Biosystems) using SYBR Green Mastermix (Applied Biosystems). Gene expression was normalised to the housekeeping genes *β2M* for untrained cells and *HPRT* for cells with a trained phenotype, and analysis was performed following a comparative Ct method. Primer sequences are listed in Table 3. Measurements were performed in duplicates.

**Table 3:** Primer sequences used for RT-qPCR

Gene	FW	RV
*b2M*	ATGAGTATGCCTGCCGTGTG	CCAAATGCGGCATCTTCAAAC
*HPRT*	CCTGGCGTCGTGATTAGTGAT	AGACGTTCAGTCCTGTCCATAA
*CAMP*	TGCCCAGGTCCTCAGCTAC	GTGACTGCTGTGTCGTCCT
*NOS2*	GCGCAGACATGATCGCCATA	CCTCACCGAACTCACC
*LAMP1*	CAGATGTGTTAGTGGCACCCA	TTGGAAAGGTACGCCTGGATG
*LGALS3*	GTGAAGCCCAATGCAAACAGA	AGCGTGGGTTAAAGTGGAAGG
*CHIT1*	ACCGTCAGACCTTTGTCAACT	CCTGTACCAGGGTTGTGAAGC
*PFKP*	CGCCTACCTCAACGTGGTG	ACCTCCAGAACGAAGGTCC
*SETD7*	ATGGATAGCGACGACGAGATG	GCAGAACCCGTGCGGTAAT
*KDM5a*	AGCCGAGTTGGGAGGAGTT	TGGACTCTTGGAGTGAAACGA

#### *In vivo* model of trained immunity in humans

##### *In vivo* and *in vitro* induction of trained immunity and *ex vivo* stimulation

*In vitro* and *in vivo* models of the 300BCG^38^ and 20BCG^39^ cohorts were performed as described elsewhere. Briefly, for *in vitro* experiments, monocytes were trained with BCG (5 μg/mL; BCG-Bulgaria strain, Intervax, Canada) and re-stimulated with LPS (10 ng/mL; serotype 055: B5). Additionally, lactate production was assessed in cell culture supernatants on day 7 of the training protocol. For *in vivo* induction of trained immunity, healthy adults were vaccinated intradermally with BCG in the left upper arm. Blood was collected in EDTA at (300BCG) baseline, two weeks post vaccination, and 90 days post vaccination, and (20BCG) baseline, two weeks, three months, and one year post vaccination. PBMCs were *ex vivo* stimulated for 24 h with heat-killed *Staphylococcus aureus* (1 × 10^6^ CFU/mL; clinical isolate), heat-killed *Mycobacterium tuberculosis* H37Rv (5 μg/mL), LPS (10 ng/mL; serotype 055: B5), or heat-killed *C. albicans* (1 × 10^6^ yeasts/mL; strain UC820). The fold increase between BCG-exposed and baseline conditions was used as a measure for magnitude of trained immunity and linked to circulating plasma IL-38 concentrations.

##### Genetic analysis – 300BCG

QTL mapping was performed as described elsewhere using genotype data and cytokine production upon stimulation.^33^^,^^59^ Both genotype and cytokine data on trained immunity responses was available for 267 and 296 individuals from *in vitro and in vivo* models of the 300CBG cohort, respectively. The log-transformed fold change of cytokine production between BCG-exposed and control conditions was used to assess the magnitude of training. Cytokine induction was mapped to genotype data using a linear regression model with age and sex as covariates. *p* < 0.05 was used as cut-off to identify suggestive quantitative trait loci (QTLs) associations affecting training responses. R-package Matrix-eQTL was used for cytokine QTL mapping.

##### Metabolomics analysis – 300BCG

Metabolite levels were measured in duplicates before BCG vaccination as described elsewhere^33^^,^^40^ and correlated to circulating plasma IL-38 concentrations (spearman correlation and direct comparison between individuals with high/low plasma IL-38 [pg/mL] by Wilcoxon matched-pairs signed rank test based on the cohort mean at visit 1). Significant hits were used for a pathway enrichment overrepresentation analysis (ORA) using a web-based server, Metabolite Set Enrichment Analysis (MSEA).^63^ Metabolites with an annotation score <50 were excluded from the analyses.

##### Proteomics analysis – 300BCG

Circulating protein abundance was assessed in plasma samples by proximity extension assay (OLINK Proteomics, Sweden).^64^ Protein expression values were log2-transformed and represented as NPX data. 92 inflammatory markers were analyzed, and reads below a detection of 75% were excluded from analyses.

##### Chromatin accessibility profiling with ATAC-seq – 300BCG

We used the previously published PBMC chromatin accessibility data (measured with ATAC-seq) of the 300BCG cohort.^38^ We downloaded the fully processed ATAC-seq count data from the Gene Expression Omnibus (GEO) stored under accession number GSE241092 and filtered the 228,191 genomic regions (mapped to the GRCh38 assembly of the human genome) to 64,065 regions located outside of the sex chromosomes and covered by at least ten reads (normalised by median library size) in at least 178 samples (corresponding to 70% of the samples for the time point with the lowest number of samples). These quality-controlled chromatin accessibility data were included for the differential accessibility analysis of 273 (visit 1), 253 (visit 2), and 254 (visit 3) PBMC samples.

##### Differential chromatin accessibility analysis – 300BCG

We used limma 3.42.0 (R 3.6.3) with the TMM normalization, “voom”, and “eBayes” functions to identify differentially accessible regions. We used the “duplicateCorrelation” function with blocking on the individual to account for inter-individual variability. Finally, we used the Benjamini-Hochberg FDR method to correct for multiple testing across all tested regions. To test for associations of chromatin accessibility with the levels of circulating IL-38, we used the following models:

$$region\;∼\;IL38+IL38:visit2+IL38:visit3+PBMC_{fractions}+blood_{draw_{time}}+sex+age+batch+TSS\_enr$$

where IL38 is a continuous variable and thus its coefficient estimates how proportional IL-38 levels are to chromatin accessibility at baseline (visit 1). The coefficients of the interaction terms IL38:visit2 and IL38:visit3 estimate how proportional the IL-38 levels are to BCG-induced changes (visit2 and visit3 relative to visit1, respectively) in chromatin accessibility. PBMC_fractions are cell fractions of monocytes, neutrophils, basophils, intermediate (CD14+CD16+) monocytes, non-classical (CD14++CD16+) monocytes, CD8+ T cells, CD4+ T cells, regulatory (CD4+CD25++) T cells, NKT cells, NK cells, and B cells. Terms blood_draw_time, sex, age, batch and TSS_enr are correction variables.

##### Enrichment analysis – 300BCG

We performed the enrichment analyses for differential chromatin accessibility separately for regions with in-creased and decreased chromatin accessibility. To this end, we ranked regions by *p*-value and tested for enrichment of the top 1,000 regions with one-sided Fisher’s exact test, implemented with *scipy* 1.4.1 (Python 3.6.10). We used the Benjamini-Hochberg FDR multiple-testing correction to adjust the enrichment *p*-values across all regions sets tested in the given analysis.

We performed gene-based pathway enrichment analyses using GO Biological Process by mapping each region to at most one protein-coding gene, and thereafter we assigned these regions to respective pathways. A region was mapped to a gene if the region’s midpoint was within 1,000 bp upstream or 500 bp downstream of the TSS, or if the region overlapped the TSS. This procedure annotated 13,002 regions (out of 64,065) to promoters of protein-coding genes.

##### Experimental murine model

Eleven-week-old WT and IL-38KO mice received b-glucan (1 mg/mouse, i.p.) or saline. On day 7, animals received LPS (5 mg/kg; *Escherichia coli*[055:B5] Sigma-Aldrich, St. Louis,MO, USA) and were sacrificed after 4 h using isoflurane. Serum was stored at −20°C. Bone marrow (BM), spleen, and whole blood were cultured without stimuli for 24 h at 37 °C, 5% CO_2_, and supernatants were stored at −20°C until ELISA. BM was stored for qPCR and epigenetic analysis as described above (Table 4).

**Table 4:** Primer sequences used for RT-qPCR and ChIP-PCR of murine samples

Gene	Target	FW	RV
*β2m*	RNA	TTCTGGTGCTTGTCTCACTGA	CAGTATGTTCGGCTTCCCATTC
*tnfa*	RNA	CAGACCCTCACACTCAGATCATCT	CCTCCACTTGGTGGTTTGCTA
*Il1b*	RNA	GCAACTGTTCCTGAACTCAACT	ATCTTTTGGGGTCCGTCAACT
*Il6*	RNA	CAAGTCGGAGGCTTAATTACACATG	ATTGCCATTGCACAACTCTTTTCT
*Pfkp*	RNA	GAAACATGAGGCGTTCTGTGT	CCCGGCACATTGTTGGAGA
*Hk2f*	RNA	TGATCGCCTGCTTATTCACGG	AACCGCCTAGAAATCTCCAGA
*Gadph*	DNA	TACTCGCGGCTTTACGGG	TGGAACAGGGAGGAGCAGAGAGCA
*chrom12*	DNA	TCTTCAAATGTTACATGGCAAATC	GTCATTTTCACTTGTTGAGAGCAG
*tnfa*	DNA	CTTGGGCCAGTGAGTGAAAG	TAGCCAGGAGGGAGAACAGA
*Il1b*	DNA	GGGAAGAGGCTATTGCTACCC	CACCACGATGACACACTTGC
*Il6*	DNA	TGCACAAAATTTGGAGGTGA	ACCCAACCTGGACAACAGAC
*Pfkp*	DNA	ACCGATAGCTTTGCCATCCC	TCTGGCGTCTCTACCTCCTC
*Hk2f*	DNA	AGCTGAGGGCCTCAAGTTTC	CTAAGCAGCTAGACCGGTCG

##### *In vivo* infection model

Nine-week-old WT mice received β-glucan (1 mg/mouse) or saline, and rhIL-38 (1 μg/mouse; 3-152aa) or saline on day 1, 2, and 3. On day 5, mice were infected with *C*. *albicans* yeast (*ATCC MYA-3573,* UC820; 1 × 10^5^/mouse) in the ocular pouch. On day 10, mice were sacrificed using isoflurane. Liver and left kidney were weighed and homogenised over a tissue disruptor. Homogenates were diluted in PBS (1×, 10×, 100×, 1000×), plated on Sabouraud glucose agar plates, and incubated overnight at 37 °C, 5% CO_2_. CFUs were counted to assess the fungal burden. Data was log-transformed and normalised to the organ weight for analyses.

##### ELISA

IL-6, IL-1β, TNF, and IFNγ (mouse and human) were measured in cell culture supernatants by DuoSet ELISA kits (R&D Systems) according to manufacturer’s instructions. IL-38 was measured in plasma samples by ELISA using the human IL-38/IL1F10 DuoSet ELISA (R&D Systems) according to the manufacturer’s instructions with extended standard curve (15.6 pg/mL as lower and 4000 pg/mL as upper limit of quantification).

##### IL-1 signaling assay

PBMCs were pre-incubated with rhIL-38 (3-152aa) (1 μg/mL) or IL-1Ra (10 μg/mL) (1 h, 37 °C, 5% CO_2_) and stimulated for 24 h with IL-1a (1 ng/mL; R&D Systems). IL-6 was measured in supernatants.

##### PBMC stimulation assay

PBMCs were pre-incubated with rhIL-38 (3-152aa) (1–100 ng/mL; 1 h) and stimulated (24 h/seven days, 37 °C, 5% CO_2_) with LPS (1 ng/mL; *E. coli* serotype O55:B5) or heat-killed *C. albicans* (1 × 10^6^ yeasts/mL; UC820). Cytokines were measured in supernatants.

### Quantification and statistical analysis

We included baseline IL-38 measures of complete cases, using values below detection as 15.6 pg/mL. IL-38 was stable over time^58^ (Friedman test; 300BCG: c^2^(3) = 3.98, *n* = 289, *p* = 0.1368; 20BCG: c^2^(4) = 2.78, *n* = 18, *p* = 0.4272). R version 4.0.3 and GraphPad Prism version 8.0.2 (GraphPad Software, CA, USA) were used. Data were analyzed by spearman correlation, one-/two-tailed Wilcoxon matched-pairs signed rank test, Kruskal-Wallis test, and one-/two-tailed, unpaired t test. Values of ∗*p* < 0.05, ∗∗*p* < 0.01,∗∗∗*p* < 0.001, and ∗∗∗∗*p* < 0.0001 were considered significant. N refers to independent experiments or animals used. Test details are indicated in figure legends.
