# Supplementary material for: Interleukin-38 is a negative regulator of trained immunity—A retrospective multi-omics study
Source: iScience. 2025 Oct 14;28(11):113758. doi: 10.1016/j.isci.2025.113758 (PMC12630041; doi:10.1016/j.isci.2025.113758)
Supplement: Document S1. Data S1–S5 [file mmc1.pdf]

iScience, Volume *28*

Supplemental information

Interleukin-38 is a negative regulator of trained immunity—A retrospective multi-omics study

Lisa U. Teufel, Vasiliki Matzaraki, Lukas Folkman, Dennis M. de Graaf, Rob ter Horst, Simone J.C.F.M. Moorlag, Jéssica C. dos Santos, Catharina M. Mulders-Manders, Thomas Krausgruber, Charles Dinarello, Mihai G. Netea, Leo A.B. Joosten, and Rob J.W. Arts

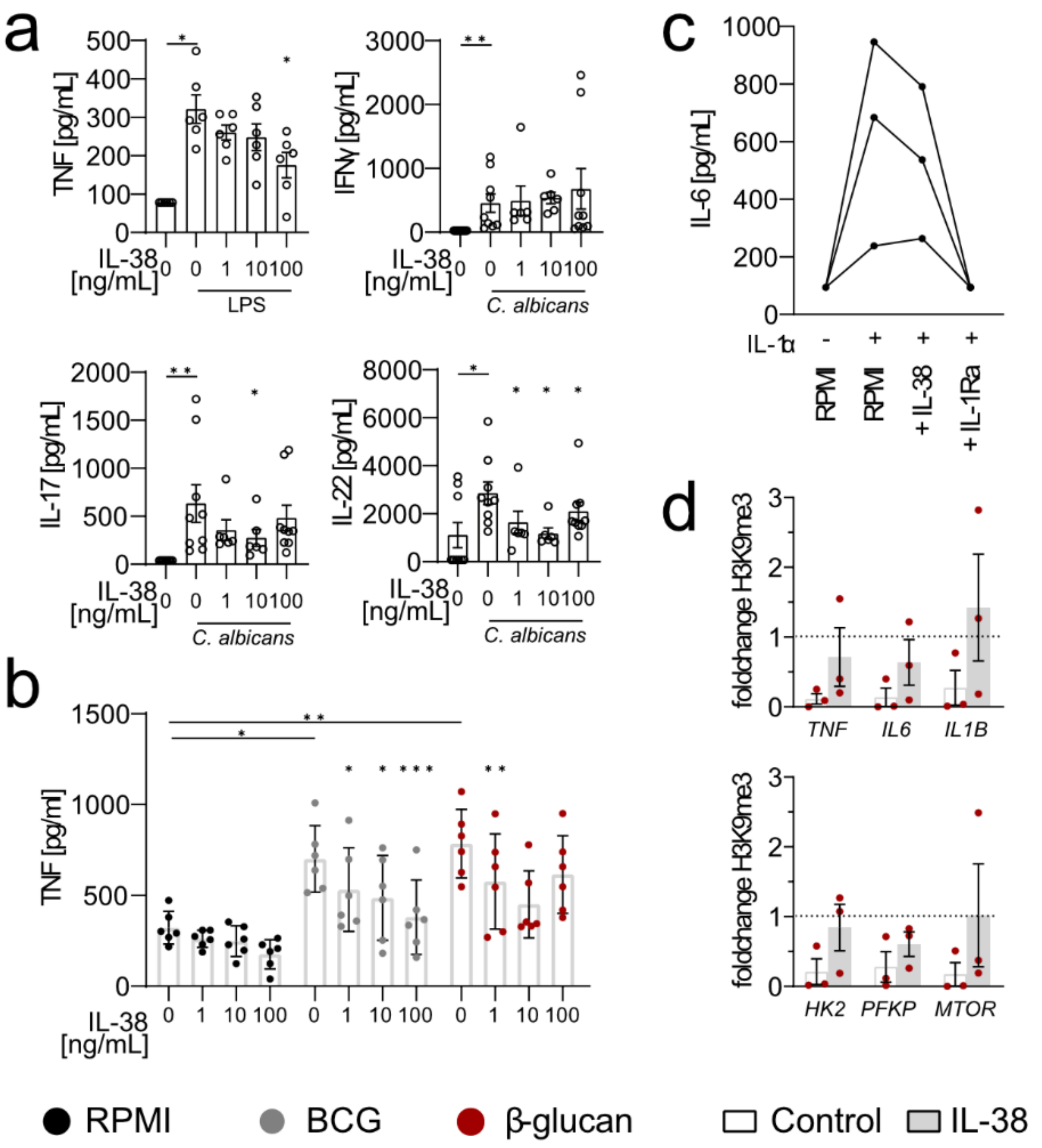

Supplemental data 1. Biological activity of rhIL-38 and concentration evaluation.

(a) Stimulation of human PBMCs with and LPS (1 ng/mL) for 24 h (TNF; n = 6) or *C. albicans* (10^6/mL) seven days (IL-22, IL-17, & IFNγ; n = 9) and varying concentrations of rhIL-38 shows a dose-dependent reduction of cytokine production. All data are presented as mean ± SEM. P values were calculated by one-tailed Wilcoxon matched-pairs signed-rank test, $^{*}p < 0.05$, $^{**}p < 0.01$.

(b) *In vitro* trained immunity assays performed on human monocytes show reduced training upon exposure to IL-38. Cells were pre-incubated with IL-38 (0 - 100 ng/mL), trained with complete medium (RPMI), β-glucan (1 µg/mL; red) and BCG (5 µg/mL; grey), and restimulated with LPS (1 ng/mL) on day six. IL-6 and TNF were measured in culture medium by ELISA after 24 h; n = 6. Data are presented as mean ± SD and were analysed by one-tailed Wilcoxon matched-pairs signed rank test, $^{*}p < 0.05$, $^{**}p < 0.01$, $^{***}p < 0.01$.

(c) Human PBMCs were pre-incubated with IL-38 (1 µg/mL) or IL-1Ra (10 µg/mL) for 1 h before stimulation with IL-1α (1 ng/mL) for 24 h. IL-6 was assessed in supernatants by ELISA; n =3. No statistical tests were performed due to the small sample size.

(d) Epigenetic signature of human monocytes (histone 3 lysine 9 trimethylation) on promoters of *HK2*, *PFKP*, *MTOR*, *TNF*, *IL6*, and *IL1B* are reduced in β-glucan (1 µg/mL; red) training which is elevated by pre-incubation with IL-38 (90 ng/mL; grey-filled); n =3. No statistical tests were performed due to the small sample size.

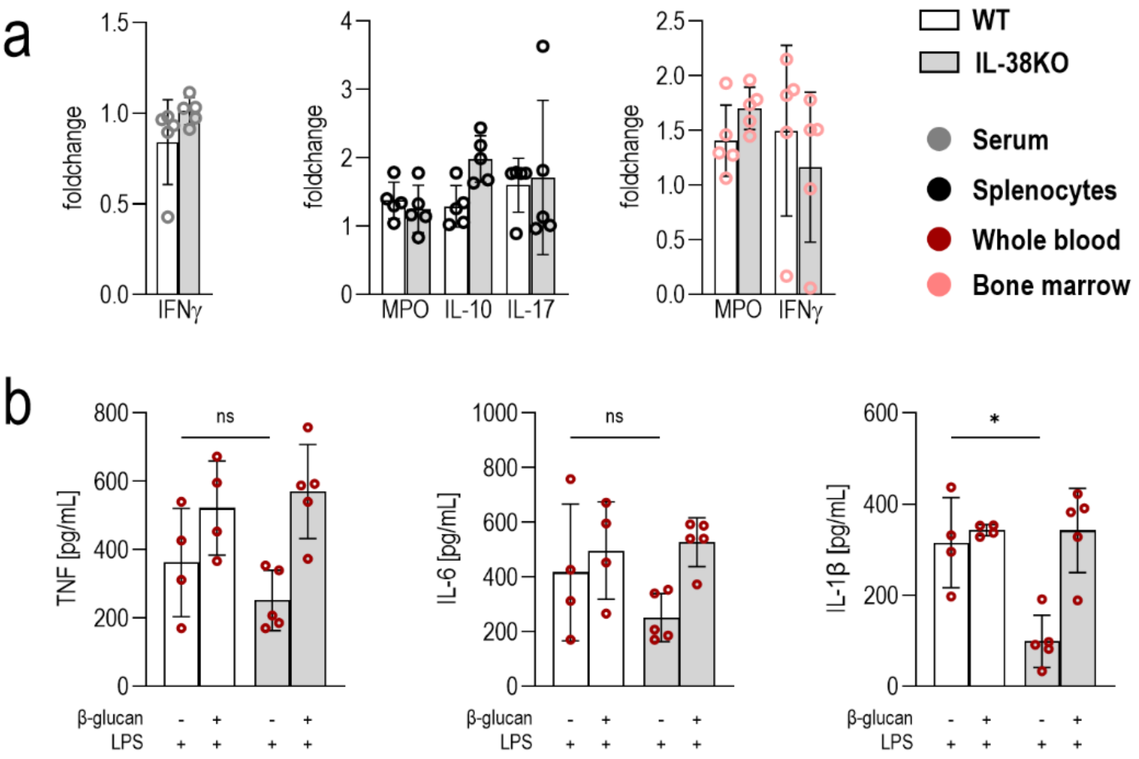

Supplemental data 4. Induction of trained immunity in WT and IL-38KO mice.

WT (white) and IL-38KO (grey) mice were trained i.p. with β-glucan (1 mg/mouse) for seven days followed by LPS (1 mg/mouse) injection and sacrifice after 4 h. Cytokine and lactate production was assessed in serum (grey) and in cell culture supernatants of bone marrow (rose) and of splenocytes (black).

(a) Training responses assessed by production of MPO, IFNγ, IL-10 were not consistently potentiated in KO animals compared to WT animals; n = 5. Data are shown as mean ± SD and analysed by one-tailed, unpaired t test.

(b) Baseline responses of whole blood against LPS are not elevated in IL-38KO mice as compared to WT animals; n = 5. Data are shown as mean ± SD and analysed by two-tailed, unpaired t test, *p < 0.05.

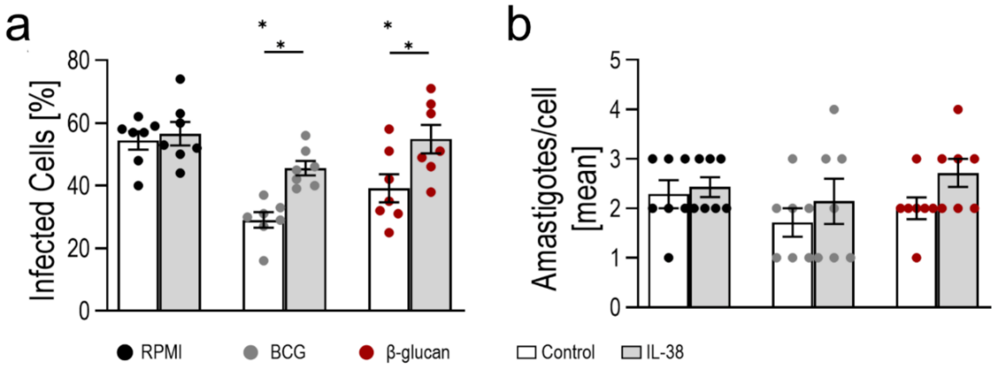

Supplemental data 5. Infection index information.

Human monocytes were infected with *L. braziliensis* for 24 h. Trained cells (β-glucan (1 µg/mL; red), BCG (5 µg/mL; grey)) show a higher killing capacity than untrained cells (black), which was opposed by pre-incubated with rhIL-38 (90 ng/mL; grey-filled). (a) Shown are infected macrophages of 100 totally counted cells in percent, and (b) mean intracellular amastigotes in all infected cells; n = 7. Data are shown as ± SEM. Data were analysed by two-tailed Wilcoxon matched-pairs signed-rank test, *p < 0.05.

Supplemental data 2. Correlation of circulating metabolites with IL-38 at visit 1.

Metabolite (bona fide)	p value	r value
Propylhydroxypentanoic acid	0.0003	-0.2071
Chenodeoxycholic acid sulfate	0.0008	-0.1918
3-Hydroxydodecanoic acid	0.0021	-0.1765
1-Arachidonoylglycerophosphoinositol	0.0031	-0.1699
1-Palmitoylglycerophosphoinositol	0.0065	-0.1567
Phenol sulfate	0.0075	-0.1539
Eriojaposide B	0.0120	-0.1447
(Z)-N-[(4-hydroxyphenyl)methyl]ethoxycarbothioamide 4''-(tri-acetylrhamnoside)	0.0163	-0.1383
C16:3	0.0166	-0.1380
D-2-Hydroxyisocaproate	0.0179	-0.1364
Glutamate	0.0194	-0.1347
6-Thioinosinic acid	0.0196	-0.1345
3''-Sialyllactose	0.0246	-0.1296
Palmitoyl glucuronide	0.0246	-0.1295
6-([13,14-dimethoxy-9-oxo-8,17-dioxatetracyclo[8.7.0.0^{2,7}.0^{11,16}]heptadeca-1(10),2(7),3,5,11,13,15-heptaen-5-yl]oxy)-3,4,5-trihydroxyoxane-2-carboxylic acid	0.0258	-0.1285
(3b,6b,8b,12a)-8,12-Epoxy-7(11)-eremophilene-6-angeloyloxy-8,12-dimethoxy-3-ol	0.0288	-0.1261
Taraxacolide 1-O-b-D-glucopyranoside	0.0288	-0.1260
1-Methoxy-1-(2,4,5-trimethoxyphenyl)-2-propanol	0.0290	-0.1258
1-Oleoylglycerophosphoinositol	0.0291	-0.1258
Phosphoserine	0.0302	-0.1250
Endoxifen O-glucuronide	0.0305	-0.1247
LysoPC(10:0)	0.0317	-0.1238
Perindoprilat	0.0328	-0.1231
Sulfolithocholic acid	0.0372	-0.1201
L-Agaridoxin	0.0376	-0.1199
Alpha-dihydroartemisinin	0.0377	-0.1198
SN38 glucuronide	0.0381	-0.1196
6-(1-carboxy-1-methylethoxy)-3,4,5-trihydroxyoxane-2-carboxylic acid	0.0382	-0.1196
(3a,5b,7a)-23-Carboxy-7-hydroxy-24-norcholan-3-yl-b-D-Glucopyranosiduronic acid	0.0418	-0.1174
6-O-Acetylaustroinulin	0.0427	-0.1169
Lagerstroemine	0.0430	-0.1167
Dulciol B	0.0430	-0.1167
Dihyroxy-1H-indole glucuronide I	0.0432	-0.1166
C19:2	0.0444	-0.1159
Doxepin N-oxide glucuronide	0.0454	-0.1154
Mesoridazine	0.0457	-0.1153
Isolimonic acid	0.0483	-0.1139
Pyruvate	0.0497	-0.1132
Cymarose	0.0494	0.1134
Acesulfame	0.0471	0.1145
Pyridoxamine	0.0406	0.1181
(6E)-7-(3-hydroxyphenyl)-1-phenylhepta-4,6-dien-3-one	0.0357	0.1211
N-(1-Deoxy-1-fructosyl)valine	0.0357	0.1211
Pantetheine	0.0357	0.1211

Dibutyl disulfide	0.0276	0.1270
Methionyl butyrate	0.0276	0.1270
Fenpropimorph	0.0274	0.1272
Mycophenolic acid	0.0272	0.1273
Ethionamide sulphoxide	0.0223	0.1317
4-Pyridoxate	0.0223	0.1317
1-(2,4,6-Trimethoxyphenyl)-1,3-butanedione	0.0222	0.1318
Anisidine	0.0198	0.1343
4-Aminohippuric acid	0.0193	0.1348
4-Guanidinobutanoate	0.0180	0.1363
Asparaginyl-Phenylalanine	0.0159	0.1389
N-(1-Deoxy-1-fructosyl)proline	0.0159	0.1389
Muramic acid	0.0106	0.1471
Methionyl-Threonine	0.0106	0.1471

Metabolites and plasma IL-38 (pg/mL) concentrations in circulation were assessed from 301 healthy volunteers. Shown are significant correlations (p values of uncorrected spearman correlations) with the corresponding spearman r value.

Supplemental data 3. Differences in abundance of circulating metabolites with IL-38 at visit 1.

Metabolite (bona fide)	p value	Log foldchange
Pseudoecgonine	0.0238	-0.0234
4-Pyridoxate	0.0153	-0.0178
Serinyl-Tryptophan	0.0346	-0.0117
Indole	0.0342	-0.0108
(±)-2-Butylthiazolidine	0.0354	-0.0107
Ximenoylacetone	0.0130	-0.0104
Lipoamide	0.0371	-0.0092
S-Acetyldihydrolipoamide	0.0456	-0.0040
4-Guanidinobutanoate	0.0242	-0.0036
Cymarose	0.0101	-0.0030
MG(0:0/14:1(9Z)/0:0)	0.0446	0.0080
Cinncassiol D2 glucoside	0.0419	0.0125
Propylhydroxypentanoic acid	0.0098	0.0136
1-Palmitoylglycerophosphoinositol	0.0025	0.0141
Dihyroxy-1H-indole glucuronide I	0.0254	0.0163
3-Hydroxycapric acid	0.0444	0.0176
1-Arachidonoylglycerophosphoinositol	0.0059	0.0191
Eriojaposide B	0.0020	0.0193
1-Oleoylglycerophosphoinositol	0.0006	0.0232
Chenodeoxycholic acid sulfate	0.0241	0.0333

Metabolites and plasma IL-38 (pg/mL) concentrations in circulation were assessed from 301 healthy volunteers. Shown are significant hits (p values of uncorrected, two-tailed Wilcox matched-pairs signed rank test) with the corresponding log2-fold change in expression comparing individuals with high (n = 99) and low (n = 220) IL-38 based on the cohort mean (135.40 pg/mL). Foldchanges below zero indicate higher metabolite concentrations in individuals with high circulating plasma IL-38.
